# Determinants of Food Choice in Athletes: A Systematic Scoping Review

**DOI:** 10.1186/s40798-022-00461-8

**Published:** 2022-06-11

**Authors:** Fiona E. Pelly, Rachael L. Thurecht, Gary Slater

**Affiliations:** School of Health and Behavioural Sciences, University of the Sunshine Coast, Sippy Downs, QLD Australia

**Keywords:** Food choice, Athletes, Scoping review, Dietary intake, Competition

## Abstract

**Background:**

The individual determinants of food choice have been extensively investigated in the general population, but there have been limited studies in athletes. A better understanding of the food making decisions can help to target interventions that lead to optimal intake for athletes’ health and performance. A scoping review will provide an understanding of the sports and settings that have been investigated, the methods and approaches to assessing food choice, as well as the factors influencing food choice.

**Objective:**

The objective of this review was to map the available evidence on the multi-faceted determinants of food choice in athletes and describe key influences impacting their choices.

Eligibility criteria.

Athletes 16 years and over from any country who engage in physical activity with the intent to be competitive. Studies were included if they reported the multi-faceted determinants of food choice as either a primary or secondary outcome. All study designs were considered.

Sources of Evidence.

This review followed the PRISMA extension for Scoping Reviews. Eleven databases including PubMed, Web of Science (Clarivate Analytics), SPORTDiscus (EBSCO), PsycNET (APA), Health Collection (Informit), CINAHL (EBSCO), the Cochrane Library, ProQuest Dissertations and Theses Global, Trove (National Library of Australia), JBI (Ovid), and Google scholar were searched between September–November 2020 and updated in March 2021.

Charting of Data

Search results were screened with selected studies extracted into a summary table established a priori by the authors. Study quality was assessed using standardised reporting tools for qualitative and quantitative research designs. The scope and quality of evidence was summarised and reported.

**Results:**

A total of 15 studies were included. Qualitative research included one research thesis and six primary research studies using both focus groups and semi-structured interviews. Quantitative research included one research thesis and seven primary research studies with cross-sectional design using different validated and non-validated survey instruments. No longitudinal or intervention studies were found. The majority of studies have been published since 2018 and conducted across multiple countries with either mixed cohorts of athletes or focused on predominately endurance or team sports. The quality of reporting was variable, particularly for qualitative research. Outcomes suggested that performance and health were relevant to athlete food choice, with varying impact of competition season, the level of experience, the culture of the sport, the cultural background or nationality of the athlete, athlete sex and the food environment.

**Conclusion:**

More research is needed on the multi-faceted determinants of food choice in different cohorts of athletes, particularly females. Future research could explore the relationship between food choice, nutrition knowledge and diet quality or the change in food choice across the phase of the seasons and through injury and illness. Use of validated measurement tools and robust reporting will enable critical interpretation of the study methods and outcomes for use in practice.

*Registration* OSF Registries: Open-ended registration 25th Sept 2020 https://doi.org/10.17605/OSF.IO/4PX2A

## Key Points

- Athletes may have adequate knowledge about healthy eating, but this may not translate into dietary intake that favours health and performance. Understanding determinants of food choice can help target interventions that lead to optimal intake for athletes’ health and performance.
- A scoping review found 7 qualitative and 8 quantitative research studies of variable quality exploring the multi-faceted factors influencing food choice for athletes. Factors specific to athletes that were based around performance or competition were evident, and these were related to the competition season, the level of experience, the culture of the sport and the nationality of the athlete.
- Future research could explore the relationship of food choice to diet, and the change in food choice across the phase of the seasons (in and out of competition) and through life events such as injury and illness. More research with female athletes is warranted.

## Introduction

The specific dietary needs for optimal health and performance of athletes vary based on the physiological demands of the sport [1]. Periodising dietary intake and tailoring eating plans to individual requirements is important for facilitating optimal nutrient intake that supports health and performance [1, 2]. There is evidence to suggest that athletes may have adequate knowledge about healthy eating, but this may not translate into dietary intake patterns that favourably influence health and performance [3, 4]. Athletes across different sports and cultures have been shown to eat inadequate amounts of the core food groups, resulting in poor diet quality [5] and subsequent compromised training adaptation [1]. A better understanding of the complexity of eating behaviours of athletes has been recommended to target interventions that lead to improved dietary intake [6].

Many of the influences on food choice applicable to the general population are also relevant to athletes. The breadth of research has originated from a variety of disciplines (for example; nutrition, psychology, marketing). An interdisciplinary framework for the factors that influence nutrition and eating across all populations was published in 2017 (The Determinants of Nutrition and Eating (DONE) [7]). Over 400 determinants of food choice were mapped into four overarching categories of individual, interpersonal environment and policy. Underpinning the framework was a systematic mapping review examining predictors of food decision making through a multidisciplinary lens [8]. The multidisciplinary perspective provides a more unified view of the determinants of nutrition and eating that have commonly been investigated in distinct disciplines or narrowed to a subsection of particular determinants and behaviours [8]. In the general population, food choice has been researched using both qualitative and quantitative study designs with the maturity of research in this field giving rise to the popularity of validated questionnaires such as the 1995 Food Choice Questionnaire (FCQ) [9].

While this research demonstrates the proliferation of studies on determinants of food choice [8], this does not specifically target populations with unique characteristics such as athletes. A 2015 narrative review on athlete food choice [10] highlighted pressure to perform, concerns over body image, the impact of exercise on hunger and appetite and exposure to unique food environments, all as having a potential role in influencing athlete food choices. The review highlighted the limited number of studies investigating the many determinants of food choice, with most studies including small numbers of athletes from specific countries and sports. Subsequently, the determinants of food choices of athletes were summarised and broadly categorised as (1) physiological and biological factors, (2) cultural background, food beliefs and preferences, (3) demographic and psychological factors, (4) education and nutrition knowledge, (5) sport and stage of competition, (6) situational influences such as cost, convenience and availability, (7) interpersonal factors including the influence of others and (8) the impact of the food service environment particularly during travel and competition [11]. This previous review was largely based around evidence that investigated the impact of a single factor on the dietary intake of an athlete. Furthermore, previous reviews have not been conducted using a systematic process for identifying all relevant studies on the topic and the quality of the studies reviewed.

Since the 2015 review, research exploring relationships between nutrition knowledge and diet quality in athletes has increased [3, 12]. While nutrition education is important, identifying the multi-faceted influences on food choice is integral to understanding the complexities of athletes’ eating behaviours. As there has been a proliferation of literature on determinants of food choice across many disciplines [7], it is of interest to scope the studies that have specifically focused on athletes. This will provide a summary of current knowledge, and will help to guide future research on this topic while concurrently assisting practitioners to understand the complexity of factors influencing the food choices of their athletes. A preliminary search of PROSPERO, MEDLINE, the Cochrane Database of Systematic Reviews and the *JBI Evidence Synthesis* was conducted and no current or in-progress scoping reviews or systematic reviews on the topic were identified. A scoping review was selected for the purpose of identifying the available evidence, to examine how research was conducted on this topic, identify key factors related to the concept and identify knowledge gaps [13]. A pragmatic paradigm [14] was employed to ensure knowledge on this topic was generated from diverse approaches and methodologies given the limited development of evidence. Inclusion of different methodological approaches can also be of benefit to guide future research direction.

The objective of this review was to collate and synthesise the evidence on the multi-faceted determinants of food choice in athletes aged 16 years or older. The following research questions guided this review: ‘What is the available evidence on the individual and interpersonal determinants of food choice in athletes?’.

The sub questions for this study were:

- What methods have been used to report on determinants of food choice in athletes?
- In what groups of athletes and sports have determinants of food choice been investigated, what are the reported outcomes on determinants of food choice and is there any relationship between demographic characteristics and food choice?
- Which studies have investigated the determinants of food choice in athletes and relationship to diet quality or intake, and what were the outcomes?
- What is the quality of reporting of studies on determinants of food choice in athletes?

## Methods

This review followed the Preferred Reporting Items for Systematic Reviews and Meta-Analyses extension for Scoping Reviews (PRISMA-ScR) [15]. A protocol for the study was developed a priori according to the Joanna Briggs Institute (JBI) methodology for scoping reviews [16] and is published on Open Science Framework [17].

### Participants–Concept–Context (PCC)

This review considered study participants specified by the authors as any individual who engages in physical activity with the intent to be competitive, 16 years and older from any sport, country, sex and performance level (professional, elite or amateur/recreational). School-based sport and studies that included children less than 16 years were excluded.

Studies that reported on the multi-faceted determinants of food choice that were measured, observed or an emerging theme of the research were included (concept). These could be reported as the primary outcome or secondary to other measures such as diet quality or intake. Studies that reported on the influence of a single determinant on food choice (for example, nutrition knowledge) were excluded. Studies that focused on specific barriers or enablers to healthy eating were also excluded unless a broader neutral question on all determinants or factors that impact food making decisions was included to align with the objective of this review. The studies could be relevant to any food environment, both in and out of a competition phase (context). Studies that investigated food choice during a race or event were excluded due to the specificity of food options and physiological impact on the body. Primary research, qualitative, quantitative, observational or intervention study designs were considered for inclusion. Studies published in peer review journals, abstract publications and research theses were considered as part of the initial screening. Early view abstracts of relevant nutrition/dietetics and sport/exercise journals were also scanned. Articles published in any language were included if they were able to be translated into English. Studies that did not meet the Participant—Concept—Context (PCC) criteria were excluded from the review.

### Search Strategy

The relevant, available databases were searched to locate published primary studies, reviews, theses, conference abstracts, and text and opinion papers. An initial search was undertaken through the SCOPUS (Elsevier B.V) database to identify articles on the topic and this was used to develop the full search strategy based on analysis of text words contained within the title, abstract, and index terms used to describe the articles were used to inform the full search strategy (provided for SCOPUS in Appendix 1). The search strategy was initially adapted from the search terms used to map predictors of food decision making to the DONE framework [8] and was refined in consultation with a librarian to ensure a robust quality process [18]. The methodological keywords from the mapping to the DONE framework were removed from the current search to ensure inclusion of both quantitative and qualitative study designs. A second search using all identified keywords and index terms was undertaken across all included databases (PubMed, Web of Science (Clarivate Analytics), SPORTDiscus (EBSCO), PsycNET (APA), Health Collection (Informit), CINAHL (EBSCO), the Cochrane Library, ProQuest Dissertations and Theses Global, Trove (National Library of Australia), JBI (Ovid), and Google scholar). The full search of all databases took place from September to November 2020 and was updated in March 2021. Finally, the reference lists of all identified reports and articles were searched for additional studies. The search was not limited by date and extended back as far as the databases allowed. Specific journals were scanned for early view abstracts based on SCImago Journal and Country Rank (SJR) subject categories (nutrition and dietetics, sports science and sports medicine). Search terms were combined using Boolean logic with the use of truncation and wildcards.

### Selection of Evidence

Records were collated and uploaded into Endnote V9.3.3 (Clarivate Analytics, PA, USA) where duplicates were removed. Following a test of the article selection process, titles and abstracts were screened by two independent reviewers (FP and RT) against the inclusion criteria. The full texts of selected citations were assessed in detail against the inclusion criteria by the same independent reviewers. Rationales for exclusion of records that did not meet the inclusion criteria were recorded. Any disagreements that arose between the reviewers at each stage of the selection process were resolved through discussion or with a third reviewer (GS). Additional records were identified through snowballing of reference lists and early view notifications.

### Data Charting Process

Data were extracted from records included in the scoping review by two independent reviewers (FP and RT) using a data extraction tool developed by the authors. The extraction tool included specific details about the participants, concept, and context, and any other information relevant to the review question. This included the title, study design and aim, participant details (sample size, athlete age, sex, level, sport and cultural background), context (country, competition season and food environment) and concept (method for reporting food choice, relationship to the food environment and any other outcomes, determinants of food choice, statistical relationship to demographics for quantitative studies and study conclusion). Any disagreements that arose between the reviewers were resolved through discussion or with a third reviewer (GS).

### Data Presentation

A tabular summary of the study details and outcomes was collated. An assessment of the quality of reporting was conducted as a means of critically appraising the extent of evidence. Critical appraisal was conducted by two of the reviewers (FP and RT) using adapted standardised reporting tools from the Enhancing the QUAlity and Transparency Of health Research (EQUATOR) library. This included the Standards for Reporting Qualitative Research (SRQR) [19] criteria for qualitative research designs and Strengthening the Reporting of Observational Studies in Epidemiology (STROBE) [20] for observational cross-sectional designs. Where members of the research team were authors of included studies, the quality appraisal was assigned to an alternative team member to ensure objectivity. Reporting of items by the authors was classified as addressed (1 point), partially addressed (0.5 point) or absent (0 points), then summed for each study as a measure of the quality of the research. Any discrepancies in the interpretation of the criteria were discussed and resolved.

## Results

**Fig. 1:**
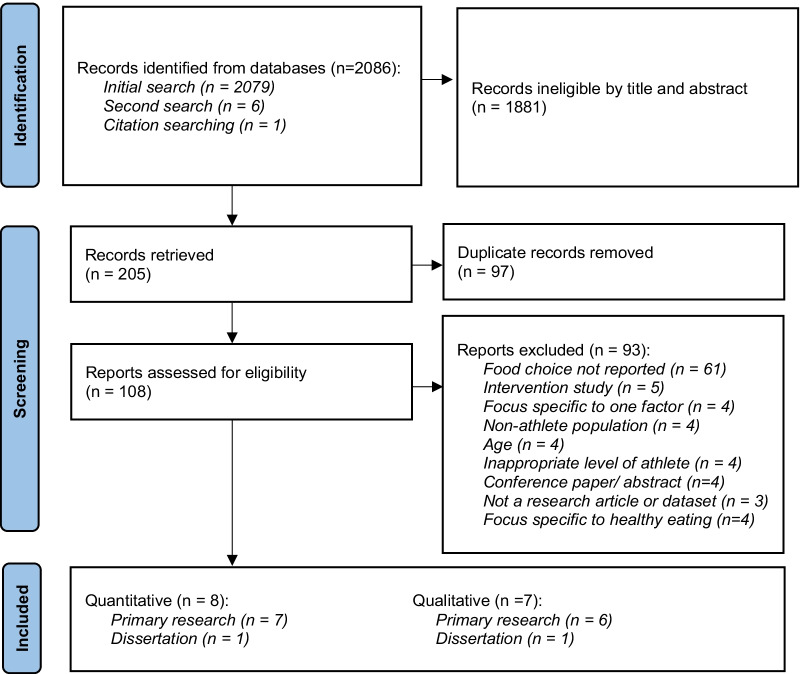
PRISMA flow diagram of the study selection process [21]

After initial identification, screening and removal of duplicates, a total 108 records were assessed for eligibility from full text. The reasons for exclusion of studies were as follows: (1) Participants were outside the age range or were not athletes, (2) Concept of food choice was not reported as an outcome or was specific to a single factor such as knowledge, (3) Context was relevant to choosing snacks during a race, or specific to healthy eating, and (4) Source of information was a review or did not contain any original data. Results of the search are presented in a Preferred Reporting Items for Systematic Reviews and Meta-analyses for Scoping Reviews (PRISMA-ScR) flow diagram [15, 21] (Fig. 1).

The final citations included 13 primary research studies [22–34], two theses [35, 36], one short conference paper [37] and three conference abstracts [38–40]. The four records identified as conference abstracts or papers were excluded. Three abstracts reported on data that aligned with one of the included primary research studies and one abstract had insufficient detail for data extraction. Narrative data were extracted from the primary research studies and theses that were included in the review (Table 1 and 2). The data were charted into two categories (1) Qualitative research design on the broader concept of food choice (one research thesis [35] and six primary research studies [22–24, 26, 33, 34], all with semi-structured interviews and predominately underpinned by grounded theory); and (2) Quantitative research design (one research thesis [36] and seven primary research studies [25, 27–32]; seven with cross-sectional observation methodology using variable survey instruments [25, 27–29, 31, 32, 36] and one cross-sectional validation study [30]. All studies were published between 2001 and 2021 with 10 published since 2018 [25–34]. No longitudinal or intervention studies were found.

**Table 1:** Data extraction: Qualitative research design

Item	2001 Smart and Bisogni [22]	2005 Robins and Hetherington [23]	2008^a^ Long [35]	2011 Long et al [24]	2018 Stokes et al. [26]	2020 Eck and Byrd-Bredbenner [33]	2021 Juzwiak [36]
Title	Personal food systems of male college hockey players	A comparison of pre-competition eating patterns in a group of non-elite triathletes	What should I eat next? Development of a theoretical model of how college-aged football players make food choices	Personal food systems of male collegiate football players: a grounded theory investigation	Perceptions and determinants of eating for health and performance in high-level male adolescent rugby union players	Food choice decisions of collegiate division I athletes: Qualitative interviews	Understanding food choices and eating practices of Brazilian and Spanish athletes in aesthetics and weight class sports
Study design	Grounded theory approach	Grounded theory approach	Grounded theory approach	Grounded theory approach	Thematic analysis of semi-structured interviews (No theory reported)	Semi-structured phone interviews (No theory reported)	Grounded theory—food choice process model
Study aim	To investigate how college athletes experienced and interpreted the multiple forces influencing their food choices	To investigate the reasons associated with the informed choices that triathletes make about their food consumption and specific eating patterns prior to competition	To understand the personal food choice process of collegiate football players	To develop a theoretical model explaining the personal food choice processes of collegiate football players	To explore perceptions and determinants of eating for health and performance in high-level male adolescent rugby union players	To improve understanding of athletes’ food-related beliefs and practices	To understand determinants of food choices and eating practices of aesthetics and weight class athletes from two countries
*Population*							
Sample size	*n* = 10	*n* = 13	*n* = 15	*n* = 15	*n* = 20	*n* = 14	*n* = 33
Sex	Male 10	Male 7 Female 6	Male 15	Male 15	Male 20	Male 5 Female 9	Not specified
Age (years)	18–23 (range)	31; 24–43 (mean; range)	Not specified	Not specified	17 ± 1; 16–18 (mean ± SD; range)	Not specified	15–42 (range)
Athlete level (as described by authors)	University – 2 freshmen, 4 sophomores, 3 juniors and 1 senior. Some with national or regional ranking	Non-elite	National Collegiate Athletic Association Division II. University – 4 sophomores, 9 juniors and 2 seniors	National Collegiate Athletic Association Division II. University – 4 sophomores, 9 juniors and 2 seniors	Highest representative -regional (60%), school (25%), national (10%), international/ age group (5%)	NCAA Division I	All competed in national and regional events 18 /33 involved in international competitions
Sport(s)	Ice hockey	Triathletes	American football	American football	Rugby union	Mixed—Swimming, track and field rowing, gymnastics, tennis, softball, volleyball	Gymnasts and martial arts
Athlete cultural background	Canada (*n* = 6), United States (*n* = 3), Eastern Europe (*n* = 1)	Not specified	Caucasian (*n* = 10), Hispanic (*n* = 3), African American (*n* = 2)	Caucasian (*n* = 10), Hispanic (*n* = 3), African American (*n* = 2)	New Zealand European (35%), Samoan (35%), Tongan (20%), Maori (10%)	Not specified	Brazil (*n* = 16) Spain (*n* = 18)
*Context*							
Country Study; author	United States; American authors	England; United Kingdom authors	United States; American authors	United States; American authors	New Zealand; New Zealand and Australian authors	United States; American authors	Brazil and Spain; Brazilian author
Competition phase	Both in- and out-of-season	Pre-competition	During training and competition season	During training and competition season	Not specified	Not specified	Not specified
Food setting/ environment	Freshmen: dormitory, university meal plan. Other participants: off-campus housing. Athletes paid for own foods, except pre-game meals that coaches /trainers arranged and ate with the team	Not specified	Athletes were responsible for their own meals usually eating with roommates or significant others Players had limited access to nutrition specialists	Athletes were responsible for their own meals usually eating with roommates or significant others Players had limited access to nutrition specialists	Not specified	Not specified	Not specified (did not include those who were provided food in live-in centres)
*Concept*							
Methods for reporting food choice	Interviews: Semi-structured face-to-face Factors that influence food choice Changes in practices between home and college, and during the season Participant observation of daily activities and informal conversation	Focus groups: Semi-structured face-to-face The reasons for food choices prior to competition 7 themes to act as probe Themes were not explicitly specified	Interviews: Semi-structured face-to-face Food/drink preferences, traditions or superstitions related to training /games Self-talk when making food selections Changes to self-talk at home or college	Interviews: Semi-structured face-to-face Food/drink preferences, traditions or superstitions related to training or games Self-talk when making food selections Changes to self-talk at home or college	Interviews: Semi-structured face-to-face Perceptions of a healthy and unhealthy diet Perceived impact of dietary intake on health and performance Barrier and enablers to healthy eating (Q: Is there anything we haven’t talked about that affects what you eat?)	Interviews: Semi–structured via phone Q: What food do you consume everyday? Why do you consume these foods? What foods do you avoid and why? Also asked about definition of healthy eating, and supplement use	Interviews: Semi-structured face-to-face Q: How do you eat during training? How do you eat in the competition phase (both questions probed with reasons for these strategies)
Relative food environment	Current /past food environments	General food environment	General food environment	General food environment	General food environment	General food environment	General food environment
*Outcome*							
Determinants of food choice	Before college: family/ home setting most influential Freshman year: new eating influences, peer influence Post freshman: independent food choice systems, beliefs about how eating related to hockey, less affected by peers Personal food systems: changing priorities between hockey, health and taste Health: ‘feeling good’, low body fat, good body image Taste: preferences, indulgence and conflicted with health Others: time, convenience, quality, quantity, variety, finances, peer influence Seasonal cycles: Off season = taste Summer = taste Dry-land training = health In season = hockey and health	32 themes in five categories): Somatic (sickness, nervous, allergy, comfort) Performance (energy, pressure, physiological need) Trust (advice, trust, nutrition knowledge, food marketing) Preferences (past experience, preferences taste, food, individual), health, convenience Routine (conditioning, routine, consistency, food planning, work-dominated eating pattern) Experience: Higher level athletes- maximizing performance, less evident with less experience Choices based on past experience for more experienced athletes	The most common themes were: Healthy Food Eat Smarter /Right No greasy food Time Money value Football players—more time planning, purchasing, and preparing meals Primary concern was to eat to compete, focusing on macronutrients and healthful foods Higher volumes of food freshman year, more college years- healthier choices Social environment - little influence from peers Physical environment - minimal influence, only the availability of choices when travelling	Theoretical model identified nine factors influencing food decisions: Time was the central influence interacting with the following: Macronutrient Money Meal themes Healthy food Food related decisions Routines Planned hydration Key outcome of food choices was to overall maintain a comfortable playing weight and to feel healthier	Three broad themes: General Influence of others (peers / family)—barriers or enablers dependent on situation Taste (barrier), cost, convenience, availability of food General/ sport-specific Mainstream and social media—barrier or enabler Physical appearance—healthy eating associated with feeling positive Sport-specific Awareness of healthy eating (game days) Timing, type, volume of food (digestion before a game) Desire to enhance sport performance Reduced healthy eating motivation during off-season Team culture a motivator for healthy eating	Prominent factors: Benefits to health and performance Avoid “unhealthy” foods – feel lethargic and inhibited ability to perform well Other factors: Cost, preference, nutrition knowledge Little difference between in and off season. More relaxed in off season, consuming fast food or dessert. Alcohol avoided during season Eat less healthy in season -when traveling, have to eat fast foods Off season, making own food- healthier	Themes included: “Perceptions about the athletic body”, “everyday food practices”, and “eating to win” Male gymnasts concerned with muscles and weight control (fear of injury) Martial arts athletes concerned with reaching fight weight Higher level of competition = stronger weight awareness Brazilian gymnasts had greater body dissatisfaction compared to Spaniards—internal and external pressure, especially from coaches Body image/weight loss was constant concern When dietary restriction is relaxed—lack of control over food with feelings of guilt Participation in social occasions is hindered by food restrictions
Conclusion	Athletes use behavioural rules and routines to manage the multiple determinants and situational nature of food and eating Food practices of athletes may be highly variable across the year of intake	Food choices and willingness for someone else to manipulate diet varied with experience/competitiveness. Food choices less important for less experienced; more experienced may have established knowledge of what works for them	Athletes have personal rules and routines informing their food choices which are based on influences from the social and physical environment	Collegiate athletes are in a highly dynamic period of life: They weigh and negotiate food choices in a new physical, social, and cultural environment	High-level male adolescent rugby players living in New Zealand have a good understanding of what eating for health and performance means	Food choices influenced by potential benefits to health and performance, availability of foods, and suggestions from sports dietitians Sports nutritionists were trusted source of nutrition information	Idealised bodies are part of the sport’s culture- impacts on beliefs/ meanings associated with eating practices. Sports-related eating practices similar in both countries, suggesting a group identity and a “sports discipline's food culture”

^a^Thesis dissertation

**Table 2:** Data extraction: Quantitative research design

Item	2014^a^ Birkenhead [36]	2018 Tesema and Mohan [27]	2018^b^ Pelly et al. [25]	2019^b^ Thurecht and Pelly [30]	2019 Pelly and Thurecht [29]	2019 Blennerhassett et al. [28]	2020^b^ Thurecht and Pelly [32]	2020 Stickler et al. [31]
Title	Nutrition knowledge, food choice motives and eating behaviours of triathletes	Determinants of athletes’ food choice motives in Ethiopian premier league football clubs	Factors influencing food choice of athletes at international competition events	Development of a new tool for managing performance nutrition: The Athlete Food Choice Questionnaire	Evaluation of athletes’ food choices during competition with use of digital images	Factors influencing ultra-endurance athletes food choices: An adapted food choice questionnaire	Key factors influencing the food choices of athletes at two distinct major international competitions	Runner’s health choices questionnaire: female collegiate cross-country runners’ perspectives on health and eating
Study design	Cross-sectional Observational	Cross sectional Observational	Cross sectional Observational	Cross-sectional Validation study	Cross sectional Observational	Cross sectional Observational	Cross-sectional Observational study	Cross sectional Observational
Study aim	To explore the nutrition knowledge, eating behaviours and factors important in the food choices of recreational triathletes compared to an age-matched group not currently participating in triathlon	To explore the key factors of food choice motives of football players in Ethiopian premier league clubs and to examine the relative importance of these factors	To investigate the influence of a selection of factors relevant to athletes that could potentially influence their food choice during two competition events	To develop and refine an Athlete Food Choice Questionnaire (AFCQ) to determine the key factors influencing food choice in an international cohort of athletes	To describe the food selection of athletes in a buffet-style dining hall setting in terms of diet quality, food variety, and volume of food. Compare to self-rating of their meal, reasons for choosing the food items, access to previous nutrition advice, and use of nutrition labelling	To assess the importance of factors that influence food choice in Ultra-endurance athletes in preparation for competition using a valid and reliable tool	To identify the key factors influencing the food choices of a diverse cohort of athletes, explore the differences in outcome between two events and describe differences across sport, history of competition and other demographic characteristics	To assess female collegiate cross-country runners’ perspectives regarding sport-related health and the factors impacting eating behaviours
*Participants*								
Sample size	*n* = 298 (164 triathletes and 134 non-triathlete)	*n* = 100	*n* = 769 (351 Delhi 2010 and 418 Melbourne 2006)	*n* = 156	*n* = 81	*n* = 101	*n* = 385 (153 Universiade and 232 Commonwealth Games)	*n* = 353
Sex	Male 152 (50%) Female 146 (49%)	Male 100 (100%)	Male 400 (52%) Female 366 (48%)	Male 64 (42%) Female 90 (58%)	Male 39 (48%) Female 42 (52%)	Males 74 (73%) Females 27 (27%)	Male 147 (41%) Female 208 (59%)	Female 353 (100%)
Age (years)	< 35–38% ≥ 35–62%	Not specified	Categorical–majority (40%) 19–24	21.5 ± 2.3; 18–28 (mean ± SD; range)	25; 15–60 (median; range)	Male 41.7 ± 8.1, Female 39.0 ± 9.6 (mean ± SD)	25 ± 7; 18–71 (mean ± SD; range)	19.5 ± 1.3 (mean ± SD)
Athlete level (as described by authors)	Active participants completing > 6 h/week of physical activity Non-triathlete: 28.4% Triathlete: 89.6%	Ethiopian Premier League	Athletes competing at the 2006 and 2010 Commonwealth Games	Athletes competing at the 2017 Universiade	Athletes competing at 2018 Commonwealth Games	Training hours per week –< 10 h (55.4%), 11-20 h (39.6%), > 20 h (3.0%),	Athletes competing at 2017 Universiade and 2018 Commonwealth Games	NCAA Divisions I (*n* = 112, 44%), II (*n* = 99, 39%), and III (*n* = 42, 17%)
Sport(s)	Triathlete (55%) (recreational, one elite and six open participants) Non-triathlete (45%)	Football (soccer)	Mixed—Power/sprint (25%), aesthetic (18%), endurance (14%), skill (13%), weight (13%), racquet (10%) and team (7%)	Mixed (17, 77.3% sports) Team (56.5%) Individual (43.5%)	Mixed (24 sports) Team (33%), endurance (24%), weight (19%), power/sprint (15%) and racket (8%)	Ultra-endurance sport: distance runner (69%), triathletes (21%), adventurers (5%) and cyclist (5%)	Mixed (29 sports) Weight (17%), power/sprint (16%), endurance (18%), racquet (8%), team (37%) and skill (11%)	Cross country running
Athlete cultural background	Not specified	Not specified	Mixed—Africa (18%), Australia/ New Zealand (9%), Canada (7%), Caribbean (10%), India/ Sri Lanka (27%), Asia Pacific (16%) and West Europe (14%)	Mixed (31, 23% countries)—Africa (13%), Europe/ United Kingdom (40%), Asia (10%), Pacific region (14%) and North and South America (24%)	Mixed (58 countries)—Africa (24%), Australia/ New Zealand (22%), British Isles (24%), Canada (10%), Caribbean (6%), Asia/Pacific (15%)	Not specified	Mixed (69 countries)—Australia/New Zealand (14%), Canada (18%), United Kingdom (8%), Africa (20%), Asia (10%), Europe/ Middle East (12%), South America/ Pacific Isles/ Caribbean (19%)	White/non-Hispanic (80%), Hispanic or Latino (9%), Black (5%), Asian (4%) and Native American or Native Hawaiian (2%)
*Context*								
Country Study; author	Australia; Australian authors	Ethiopia; Indian authors	India and Australia; Australian authors	Taiwan; Australian authors	Australia; Australian authors	England; United Kingdom authors	Taiwan and Australia; Australian authors	United States; American authors
Competition phase	Pre-competition Recruited in the 3 months prior to the Noosa 2012 Triathlon	Not specified	In competition	In competition 34% event/s not finished 66% event/s completed	In competition 69% event/s not finished 31% event/s completed	Pre-competitive assorted competition events not specified	In competition 56% event/s not finished 44% event/s completed	Not specified
Food setting/ environment	Not specified	Not specified	Live in village, buffet style, food provided, self-select, no cost	Live in village, buffet style, food provided, self-select, no cost	Live in village, buffet style, food provided, self-select, no cost	Not specified	Live in village, buffet style, food provided, self-select, no cost	Not specified
*Concept*								
Methods for reporting food choice	Questionnaire: Importance five-point Likert scale Adapted from the Food Choice Questionnaire (FCQ)–Revised version by Lockie et al. 2002. Pilot tested (*n* = 16) Factors rated: health, weight, performance, mood, convenience, sensory appeal, natural content, price, familiarity, animal welfare, environmental protection, political values and religion	Questionnaire: 13 food choice factors Note: appears to be the adapted FCQ used in Birkenhead’s thesis	Questionnaire: Importance five-point Likert scale Factors rated: nutrient content, visual appearance, smell, familiarity, stage of competition, time of day, proximity to entrance, presence of teammates, presence of coach	Questionnaire: Frequency five-point Likert scale Groups: sensory attributes, convenience and access to food, usual eating practices, food production and marketing, emotional influences, food and health awareness, nutritional attributes of the food, performance, influence of others, and situational influences	Questionnaire: Open ended questions Self-reported influences on their food selection. Answers were analysed into themes based on the categories Athlete Food Choice Questions (AFCQ)	Questionnaire Phase 1: Pilot tested 84-item questionnaire. Adapted Food Choice Questionnaire for ultra-endurance athletes (U-FCQ) Phase 2: U-FCQ Importance seven-point Likert scale Factors rated: access, convenience, mood, sensory appeal, ethical concern, allergy, health, physique, trust, somatic, event and familiarity	Questionnaire: Frequency five-point Likert scale AFCQ and 11 additional items; availability, cost, convenience, eating location, doping concerns, gut comfort, hunger, the meal, busy schedule, and medical conditions and food allergies Open ended questions asked about additional factors that may influence food choices	Questionnaire: Runner’s Health Choices Questionnaire Response options (no, minimal, moderate or high impact and neutral/ don’t know) Rate how much of an impact you feel the following 13 factors have on your choice of: Overall diet; and, Daily meal decisions
Relative to food environment	General food environment over the past 3 months	General food environment	Current food environment	General food environment	Current food environment–specific to current meal	General food environment and competition	General food environment	General food environment
Other outcomes	General Nutrition Knowledge Questionnaire: Three Factor Eating Questionnaire	N/A	N/A	Phase of competition and competition history	Sources of nutrition information, dietary regimens, self-rating of food selection Digital images of meals Quantitative and qualitative nutritional analysis	Dietary restrictions (habitual, pre-competition and during competition)	Phase of competition and competition history	Factors that impact overall health and running performance, sources of nutrition information
*Outcome*								
Determinants of food choice	Health Performance Price Sensory appeal Natural content Convenience Weight Mood Familiarity Animal welfare Political values Environmental protection	Health Weight control Price Sensory appeal Environmental protection Natural contents Familiarity Religion Mood Convenience Political value Animal welfare	Nutrient composition Stage of competition Familiar food Time of day Smell Visual appearance Teammates Proximity to entry Coach	Nutritional attributes Emotional influences Food /health awareness Influence of others Usual eating practices Weight control Food values and beliefs Sensory appeal Performance	Nutritional attributes (macronutrient content of food, content in meal) Sensory factors Performance Usual eating practices (food preference or familiarity) Influences reported in smaller numbers: Food/health awareness, emotional influences, weight control, influence of others Physiological reasons (gut comfort, hunger, satiety) Other factors (weather/climate, availability, health condition)	Equal mean rating—Event, somatic Sensory Health Equal mean rating Nutrients, physique trust Feelings Access Convenience Time Important: Provide me with energy’, ‘do not cause me gastrointestinal discomfort’ ‘Nutritious’ ‘Tastes good’, ‘are good quality products’ and ‘keeps me healthy’	Performance Sensory appeal Food and health awareness Weight control Top additional items: Hunger Time of day Gut comfort Convenient to prepare Unique factors: Preferences, exploratory eating, competition phase, weather, food safety and transport	Greatest proportion of high impact responses: Overall diet: Enjoyment of food Makes you feel healthy Athletic performance enhancement Health condition Daily food choices: Practice/race that day Choices in the cafeteria Creating a balanced diet Time to prepare meals
Relationship to other variables	More important: Sex: Females—weight and natural content Sport: Triathletes -performance and price. Non-triathletes—environmental protection, political values and animal welfare Exercise: Active males—weight control Less important Active individuals—sensory appeal	Setting: Differences in price, health, fitness and performance, weight control, animal welfare, sensory appeal and religion between football clubs Nationality: Foreign players more affected by health and natural content Ethiopian players more affected by price, environmental protection and religion Football club and nationality: Associated with political values and familiarity Education: Significant mean difference for convenience, weight control, animal welfare and religion factors	More important Setting: Delhi—Coach and teammates, visual appearance and time of day Sex: Females—Smell and familiarity Sport: Weight category and endurance—Stage of competition and nutrient composition Weight category—coach Culture: Indian and Asia Pacific—teammates and coach more than Canada, Australia and West Europe	Intercorrelations between performance and both nutritional attributes of the food and weight control	Meals in general lacked fruit, dairy and included discretionary foods Athletes’ self-rating of food was 8–10 Positive correlation between age and self-rating Young athletes rating meal as poorer	N/A	Experience: Performance more and emotional influences less in Commonwealth Games than Universiade Age: Younger athletes more frequently reported available money as an influence Sport: Food and health awareness, nutritional attributes of food and weight control more frequently reported by weight category athletes Culture: Food values and beliefs and doping concerns more frequently reported from non-western countries	N/A
Conclusion	Athletes in this study placed high importance on performance and health when making food choices but were less concerned about factors related to ethical issues and religion	Factors which can affect players’ food choices can differ based on the athlete’s playing club and nationality	Unique influences on food choices of athletes in a competition environment, which is influenced by their sport and cultural background	This research resulted in a questionnaire (AFCQ) that included factors specific to athletic performance and the sporting environment	Findings suggest that athletes may be more focused on the quantity of macronutrients rather than the quality of food and are influenced by a range of factors, even if having had previous nutrition advice	The study produced a questionnaire with evidence of reliability. The questionnaire may be used to assess the factors that influence food of ultra-endurance athletes during periods of high-volume training and competition	More experienced athletes may be more influenced by performance and nutrition, and less so by their emotions, Competition phase appears to have a modulating effect on food choice motives	A variety of intrinsic and extrinsic factors influence female collegiate cross-country runners’ health status and eating choices

^a^Thesis dissertation

^b^Related abstracts/conference proceedings: Thurecht and Pelly 2018 [40], Thurecht 2020 [39] Pelly F et al. 2006 [37].

Additional unrelated abstract: Tuğal and Bilgiç 2019 [38].

Five studies (four quantitative [25, 29, 30, 32] and one qualitative [33]) involved athletes across multiple sports. Single sports that were investigated included endurance (*n* = 4; triathlon, cyclists, distance runners and adventurers, cross country running) [23, 28, 31, 36], team (*n* = 5; soccer [27], ice hockey [22], American Football [24, 35], rugby union [26]) and aesthetic (*n* = 1; gymnastics and martial arts [34]) sports. Of the quantitative studies, three were conducted during competition [29, 30, 32], two pre-competition [28, 36], and two were not specified [27]. The qualitative studies consisted of one pre-season [23], three not specified [26, 33, 34], and three conducted both in and out of competition [22, 24, 35].

Athletes’ home country varied with mixed cohorts from multiple locations (*n* = 4) [25, 29, 30, 32] and those specific to individual countries (USA *n* = 5 [22, 24, 31, 33, 35], Australia *n* = 1 [36], New Zealand *n* = 1 [26], Brazil and Spain *n* = 1 [34], Britain *n* = 2 [23, 28], Ethiopia *n* = 1 [27]. Five studies [23, 27, 28, 33, 36] did not report the cultural background of the athletes participating in the study. The mixed cohort studies [25, 29, 30, 32] were conducted at international multisport competitions and reported participants from 31 to 69 different countries.

All qualitative studies reported on emerging themes on determinants of food choice relevant to the sample of athletes. The outcomes of the qualitative studies suggested health [22, 24, 26, 33, 35] and competition performance [23, 26, 33–35] were important motives influencing food choice, but this was impacted by seasonal differences [22, 26, 33], athlete experience [22, 23, 34], and constraints on time [22, 24, 35] and money [24, 35]. More experienced athletes were reported to be less influenced by others and more focused on performance [22, 23, 34]. The quantitative studies reported determinants of food choice ranked from highest to lowest priority, or as a list, and in relationship to other characteristics of the cohort. Determinants that occurred across multiple studies included health [28–31, 36], performance [29–32, 36], nutritional attributes/composition [25, 28–30], familiarity/usual eating [25, 27, 29, 30, 36], sensory factors [25, 27–30, 32, 36], convenience [27, 28, 32, 36], mood/feelings [27, 28, 30, 36] and weight control [27, 30, 32, 36]. In the mixed cohort studies that explored relationships to the characteristics of their sample, sex [25, 36], sport [25, 32, 36], age [32], culture/nationality [25, 27, 32] and setting [25, 27] influenced the priority given to specific factors. Only one study explored the relationship between food choice and diet quality [29]. A summary of the determinants of food choice from all studies (41 in total) has been grouped into eight broad categories adapted from previous reviews [10, 11] and the DONE framework [7] and included in Table 3.

**Table 3:** Determinants of food choice grouped according to broad categories*

Category	Determinant / outcomes
Physiological factors	Sensory (e.g. taste) Illness/health condition Food allergy Gut comfort
Cultural background, food beliefs and preferences	Preference Familiarity Animal welfare Political values Environmental/sustainability Cultural background/beliefs
Socio- demographic	Age Sex Sport Nationality
Psychological factors	Nervousness Body image Guilt Mood Enjoyment
Health and nutrition perceptions	Trust Healthiness Natural content Nutritional content Food quality
Sport and stage of competition	Season/phase Experience Playing weight/weight control Timing in regard to competition Enhanced performance
Situational influences	Time to eat Routine Cost Convenience Social media Marketing Travelling Accessibility Exploratory eating Weather Food safety
Interpersonal factors including the influence of others	Teammates/peers Family

^*^Categories adapted from previous reviews [10,11] and DONE framework [7].

The quality assessment resulted in a total score for the qualitative studies that ranged from 10 to 20 out of a total of 21 (median 16.5), and the quantitative studies ranging from 8 to 22 out of 32 (median 21.5) (Table 4 and 5). No study reported on every item in either of the quality reporting tools.

**Table 4:** Quality assessment of qualitative studies using SRQR criteria [20].

	Brief description	Smart et al. [22]	Robins et al. [23]	Long [35]^a^	Long et al. [24]	Stokes et al. [26]	Eck et al. [33]	Juzwiak [34]
1	Title				*		*	
2	Abstract	*	*	*	*	*	*	*
3	Problem formulation	*	*	*	*	*	*	*
4	Purpose / research question	*	*	*	*	*	*	*
5	Qualitative approach/ research paradigm	P	P	*	*			*
6	Researcher characteristics/ reflexivity	*			*			P
7	Context	*		*	*			*
8	Sampling strategy	*		*	*	*		*
9	Ethical issues	*		*	*	*	*	*
10	Data collection methods	*		*	*	*	*	*
11	Data collection instruments / techniques	*	P	*	*	*	*	*
12	Units of study	*	*	*	*	*	*	P
13	Data processing	*		*		*	*	*
14	Data analysis	*	*	*	*			*
15	Techniques to enhance trustworthiness	*		*	*	*		*
16	Synthesis and interpretation	*	*	*	*	*	*	*
17	Links to empirical data	*	*	*	*	*	*	*
18	Integration with prior work/ implications / transferability/ contribution	*	*	*	*	*	P	*
19	Limitations	*	*	P	*	*	P	*
20	Conflict of interest				*		*	
21	Funding				*	*	*	*
	Total score	**17.5**	**10**	**16.5**	**20**	**15**	**14**	**18**

Bold values indicate total score of quality based on the sum of the number of items that meet the reporting criteria for each study

* = addressed by authors; *P* = partially addressed by authors

^a^PhD thesis

1. Concise description of the nature and topic of the study identifying the study as qualitative or indicating the approach (e.g., ethnography, grounded theory) or data collection methods (e.g., interview, focus group) is recommended

2. Summary of key elements of the study using the abstract format of the intended publication; typically includes background, purpose, methods, results, and conclusions

3. Description and significance of the problem/phenomenon studied; review of relevant theory and empirical work; problem statement

4. Purpose of the study and specific objectives or questions

5. Qualitative approach (e.g., ethnography, grounded theory, case study, phenomenology, narrative research) and guiding theory if appropriate; identifying the research paradigm (e.g., postpositivist, constructivist/interpretivist) is also recommended; rationale

6. Researchers’ characteristics that may influence the research, including personal attributes, qualifications/experience, relationship with participants, assumptions, and/or pre-suppositions; potential or actual interaction between researchers’ characteristics and the research questions, approach, methods, results, and/or transferability

7. Setting/site and salient contextual factors; rationale

8. How and why research participants, documents, or events were selected; criteria for deciding when no further sampling was necessary (e.g., sampling saturation); rationale

9. Documentation of approval by an appropriate ethics review board and participant consent, or explanation for lack thereof; other confidentiality and data security issues

10. Types of data collected; details of data collection procedures including (as appropriate) start and stop dates of data collection and analysis, iterative process, triangulation of sources/methods, and modification of procedures in response to evolving study findings; rationale

11. Description of instruments (e.g., interview guides, questionnaires) and devices (e.g., audio recorders) used for data collection; if/how the instrument(s) changed over course of the study

12. Number and relevant characteristics of participants, documents, or events included in the study; level of participation (could be reported in results)

13. Methods for processing data prior to and during analysis, including transcription, data entry, data management and security, verification of data integrity, data coding, and anonymization/deidentification of excerpts

14. Process by which inferences, themes, etc., were identified/ developed, including the researchers involved in data analysis; usually references a specific paradigm or approach; rationale

15. Techniques to enhance trustworthiness and credibility of data analysis (e.g., member checking, audit trail, triangulation); rationale

16. Main findings (e.g., interpretations, inferences, and themes); might include development of a theory or model, or integration with prior research or theory

17. Evidence (e.g., quotes, field notes, text excerpts, photographs) to substantiate analytic findings

18. Short summary of main findings; explanation of how findings and conclusions connect to, support, elaborate on, or challenge conclusions of earlier scholarship; discussion of scope of application/generalizability; identification of unique contribution(s) to scholarship in a discipline or field

19. Trustworthiness and limitations of findings

20. Potential sources of influence or perceived influence on study conduct and conclusions; how these were managed

21. Sources of funding and other support; role of funders in data collection, interpretation, and reporting

**Table 5:** Quality assessment of quantitative studies using STROBE criteria [20].

STROBE criteria		Birkenhead [36]^a^	Tesema et al. [27]	Pelly et al. [25]	Thurecht et al. [30]	Pelly et al. [29]	Blennerr-hassett et al. [28]	Thurecht et al. [32]	Stickler et al. [31]
*Title and abstract*									
1	Design in title			*	*	*	*	*	*
2	Informative abstract	*	*	*	*	*	*	*	*
*Introduction*									
3	Rationale	*		*	*	*	*	*	*
4	Specific objectives	*	*	*	*	*	*	*	*
*Methods*									
5	Study design	*		*	*	*	*	*	*
6	Setting	*	*	*	*	*	*	*	*
7	Participants	*		*	*	*	*	*	*
8	Variables	*		*	*	*	*	*	*
9	Data source	*		*	*	*	*	*	*
10	Bias								
11	Study size			*	*			*	*
12	Quantitative variables	*		*	*	*	*	*	*
13	Statistical methods	*	*	*	*	*	*	*	*
14	Subgroups & interactions	*	*	*	*	*	*	*	
15	Missing data								
16	Sampling strategy	NA	N/A	NA	N/A	N/A	N/A	NA	N/A
17	Sensitivity analysis								
*Results*									
18	Participants	*		*	*	*	*	*	*
19	Non-participation								
20	Flow diagram								
21	Descriptive data	*		*	*	*	*	*	*
22	Missing data	*			N/A	N/A	*		*
23	Outcome events	*		*	*	*	*	*	*
24	Confounders								
25	Category boundaries	N/A	N/A	NA	N/A	N/A	N/A	NA	*
26	Risk	N/A	N/A	NA	N/A	N/A	N/A	NA	N/A
27	Other analyses	*	*	*	*	*	*	*	N/A
*Discussion*									
28	Key results	*	*	*	*	*	*	*	*
29	Limitations	*		*	*	*	*	*	*
30	Interpretation	*	*	*	*	*	*	*	*
31	Generalisability	*			*	*	*	*	*
Other *information*									
32	Funding			*	*	*	*	*	*
	Total score	**20**	**8**	**20**	**22**	**21**	**22**	**22**	**22**

Bold values indicate total score of quality based on the sum of the number of items that meet the reporting criteria for each study

* = addressed by authors; *P* = partially addressed by authors; N/A = not applicable

^a^Masters thesis

1. Indicate the study’s design with a commonly used term in the title or the abstract

2. Provide in the abstract an informative and balanced summary of what was done and what was found

3. Explain the scientific background and rationale for the investigation being reported

4. State specific objectives, including any prespecified hypotheses

5. Present key elements of study design early in the paper

6. Describe the setting, locations, and relevant dates, including periods of recruitment, exposure, follow-up, and data collection

7. Give the eligibility criteria, and the sources and methods of selection of participants

8. Clearly define all outcomes, exposures, predictors, potential confounders, and effect modifiers. Give diagnostic criteria, if applicable

9. For each variable of interest, give sources of data and details of methods of assessment (measurement). Describe comparability of assessment methods if there is more than one group

10. Describe any efforts to address potential sources of bias

11. Explain how the study size was arrived at

12. Explain how quantitative variables were handled in the analyses. If applicable, describe which groupings were chosen and why

13. Describe all statistical methods, including those used to control for confounding

14. Describe any methods used to examine subgroups and interactions

15. Explain how missing data were addressed

16. Describe analytical methods taking account of sampling strategy

17. Describe any sensitivity analyses

18. Report numbers of individuals at each stage of study, e.g., numbers potentially eligible, examined for eligibility, confirmed eligible, included, completing follow-up, and analysed

19. Give reasons for non-participation at each stage

20. Consider use of a flow diagram

21. Give characteristics of study participants (e.g., demographic, clinical, social) and information on exposures and potential confounders

22. Indicate number of participants with missing data for each variable of interest

23. Report numbers of outcome events or summary measures

24. Give unadjusted estimates and, if applicable, confounder-adjusted estimates and their precision (eg, 95% confidence interval). Make clear which confounders were adjusted for and why they were included

25. Report category boundaries when continuous variables were categorized

26. Consider translating estimates of relative risk into absolute risk for a meaningful time period

27. Report other analyses done, e.g., analyses of subgroups and interactions, and sensitivity analyses

28. Summarise key results with reference to study objectives

29. Discuss limitations of the study, taking into account sources of potential bias or imprecision. Discuss both direction and magnitude of any potential bias

30. Give a cautious overall interpretation of results considering objectives, limitations, multiplicity of analyses, results from similar studies, and other relevant evidence

31. Discuss the generalisability (external validity) of the study results

32. Give the source of funding and the role of the funders for the present study and, if applicable, for the original study on which the present article is based

## Discussion

The purpose of this scoping review was to examine the available evidence on the multi-faceted individual and interpersonal determinants of food choice in athletes with a focus on participant characteristics, methods used to collect data, study outcomes and the overall quality of the evidence. While research on this topic spans over the past 20 years, most studies were conducted during the past five years. Studies have investigated food choices of athletes in a variety of sports and countries through a mixture of both quantitative and qualitative methods. The majority of earlier studies were qualitative and exploratory in nature and conducted with smaller samples of predominately male collegiate athletes. In general, the outcomes of the qualitative studies reported that the social and physical food environment, sport or team culture, the phase of competition and experience of the athlete impacted food choice. This is supported by outcomes from more recent qualitative research which suggests that the high-performance environment and athletes’ emotional state may impede adherence to nutrition guidance [6].

The outcomes of the quantitative studies demonstrated that nutritional attributes of the food and performance were considered when making a food choice and, in most cases, these were high priorities. This was measured across multiple sports and in various settings in and out of competition. Weight control was raised as a higher priority impacting food choice for female triathletes [36], and athletes in weight category sports [25, 30]. This aligns with the qualitative study by Juzwiak (2021) [34] that investigated food choice in weight class athletes from Brazil and Spain, and found predominant themes focused on the food culture of the sport related to body image and weight, and with the study by Long [24] which found a comfortable playing weight was factored into food choices of male American Football players. A focus on body image and the pressure to maintain an ideal physique aligns with qualitative studies that have specifically focused on barriers to health or performance-based eating [6, 41].

Cultural background and nationality also appeared to be influential in terms of food choice, but the impact on food choice varied across studies with influence of others [25], food values and beliefs [32], doping [32], political values [27], religion [27], price [27], environmental protection [27] and familiarity [27] all reported. In general, there was significant variability in the relationship of demographic and sporting characteristics of athletes and inconsistency in priority ranking of determinants of food choice in the quantitative studies. Despite this, there was consistency in the reported determinants of food choice across the limited number of studies on this topic.

The broader relationship of food choice to diet quality or intake of athletes was not found through the search, although Pelly and Thurecht reported on the quantitative and qualitative dietary analysis of a single meal and the reasons the athletes chose this meal [29]. In this case, athletes reported choosing food based on the nutritional attributes of the food, sensory factors performance or usual eating practices, but in general, the meals lacked fruit and dairy and included discretionary foods. Interestingly, athletes self-rated their meal choice in relation to their performance needs as an eight (10 = excellent), but this was dependent on age with younger athletes rating their meal selection less highly [29]. This may be due to lack of experience which impacts confidence in food choice. The determinants of food choice in athletes can provide a valuable understanding of the disjointed relationship between nutrition knowledge and appropriate dietary intake for health and performance. Research on this topic can be a useful strategy to raise awareness and target education of athletes.

A sub-question of this review was to examine the methods used to report on the determinants of food choice. The earlier qualitative studies focused on developing theory [22–24, 35] on the multiple influences of food choice, and in particular the process of the food choice decision. The foundation of qualitative research appears to have led to the more recent use of survey instruments in quantitative cross-sectional studies, as a means for examining the relationships of the multi-faceted aspects of food choice and comparison to athlete characteristics. We found the initial quantitative studies used an adapted version of a validated tool developed for the general population, The Food Choice Questionnaire (FCQ) [7, 27, 36], or a non-validated survey applied in two settings [25]. Three survey instruments specific to athletes were published during 2019–2020. The instruments include the Athlete Food Choice Questionnaire (AFCQ) [30], the Adapted Food Choice Questionnaire for ultra-endurance athletes (U-FCQ) [28] and Runner’s Health Choices Questionnaire (RHCQ) [42]. Validation and reliability in survey instruments is important and within this conceptual space where no objective criterion measure is available to truthfully know what influences food decisions, using appropriately developed and tested instruments is imperative. Multiple psychometric tests are advised in the development of new instruments within health, social and behavioural research to establish validity and impart confidence in a new instrument [43]. The AFCQ is a broadly applicable instrument developed and validated in two mixed sport and cultural background samples of high-performance athletes [30, 44]. Development via exploratory factor analysis informed face and content validity, while confirmatory factor analysis in an independent sample confirmed the consistency of the AFCQ’s factorial structure [30, 44]. Construct validity was established with duplicate measures of discriminant and convergent validity, achieving acceptable thresholds for nine and six factors, respectively. Cronbach’s alpha measured reliability with seven factors exceeding the accepted standard (> 0.7) and two exceeding a tolerable 0.6 threshold [44].

The U-FCQ adapted the FCQ and through pilot testing (*n* = 19) refined the questionnaire items via an interpretive process simulating an exploratory factor analysis [28]. The development provides support for face and content validity, while acceptable reliability was evidenced for all 11 factors via Cronbach’s alpha and eight factors via test–retest analysis. The RHCQ measures the influence of 13 single-item factors on overall dietary choice and daily food choices.[42] Development included expert review (*n* = 3) and pilot testing with the target population (*n* = 26) to establish face and content validity; reliability, however, was not examined [42]. To date, the AFCQ has undertaken the most extensive examination of reliability and validity [30, 44]. This process is encouraged for recently published instruments.

To better understand the strength of evidence on the topic, the quality of reporting each study was critically assessed. Qualitative studies varied in their quality with Smart [22], Long [24] and Juzwiak [34] being the most comprehensive. More stringent requirements for reporting of qualitative study design over the past 10 years are likely responsible for the increased quality of reporting in recent studies. In particular, transparent reporting and rationale for the research paradigm (3 out of 7 studies) and the researcher characteristics that may influence the study (3.5 out of 7 studies) were poorly reported. In the case of the studies that included reflexivity, the researchers declared that they were predominately from a health or sport background which explains the focus on nutritional aspects of the food and performance factors as emerging themes. All the quantitative studies with the exception of one (Tesema et al.) [27] were of a similar quality in terms of reporting the details of the study but were less transparent on how they arrived at the sample size, how they addressed any missing data, and reasons for non-participation which may have introduced a level of bias in the results. Furthermore, if the included studies were mapped to the respective discipline area as per the DONE framework [8], all were based on a nutrition or sports science, psychology or health paradigms suggesting that the outcomes may be limited by the approach or theory underpinning the research. Assessing the quality of available evidence supports replication of good quality study designs and reporting practices plus enables critical interpretation of the study methods and outcomes for use in practice.

Future research on this topic is needed to better understand the priority various cohorts of athletes from a range of sports and cultures place on different factors when making food choice decisions. It would be of benefit to explore the relationship between food choice, nutrition knowledge and diet quality directly. We recommend that researchers use a survey that is validated for the purpose of exploring the multi-faceted determinants of food choice in this situation. While the AFCQ has currently undergone the most robust validation with multiple groups of athletes, further testing of reliability and application with specific sports and in different cultures is warranted. Further exploration of actual dietary intake across multiple days followed by the AFCQ could provide insight into the reasons for meal and snack selection. Investigation of the change in food choice longitudinally across the phase of the seasons (in and out of competition) and through life events such as injury and illness, retirement or changes in social situations would also be of interest. Interventions targeted at factors influencing athlete food choice to facilitate behaviour change as well as factors influencing food choice for specific sports and in different cultural contexts could be investigated.

The research reported in this review focused on individual or interpersonal factors influencing food choice and did not specifically examine the food environment or policies impacting food making decisions for the athlete. At a broader level, the availability and cost of specific foods will often underpin individual food choice, as will marketing and promotional campaigns [45] and situational factors such as catered food during competition and travel [11]. A study of athletes’ opinions of the food provided during a major competition has shown that the availability of appropriate food is driven by cultural acceptance, and this may impact food choice [46]. The relationship between the food environment and individual food choices of athletes across different countries, regions and sociocultural contexts would be of value as this has been identified as an area for further research in general populations [47]. The interplay between physiological function (for example; appetite, gut function, brain regulation), psychological factors, food beliefs, knowledge and skills, and the food environment relevant to athletes needs further investigation. As the earlier qualitative research informed the development of the quantitative questionnaires, it would also be of interest to conduct more studies with female athletes as only one quantitative study by Stickler (2020) [31] specifically focused on females. Sex specific issues that impact food choice could be further explored using qualitative research that specifically explored eating behaviours.

There are limitations associated with this scoping review. It is feasible that not all studies were found during the search, but it is unlikely that this would be a major impact on the determinants of food choice reported in this review because outcomes were consistent across studies. A decision was made to exclude any study that investigated an individual or pair of determinants in isolation (for example, the impact of taste or smell on athlete food choice). A multi-faceted approach was taken to explore the relationship between different determinants in the broader context of food choice. Studies that investigated eating behaviours such as barriers or enablers to healthy eating or good nutrition were excluded as the aim was to explore the interplay between all factors that potentially influence food choice. For example, an athlete may not indicate that taste is an influence on their food choice if only asked about the barriers to healthy eating. One study (Stokes et al.) [26] included a generic question of food choice as part of their questioning on barriers and enablers to healthy food intake, and thus this study was included in this review. There is a chance, albeit small, that additional studies with questions of this nature were not identified through our search.

## Conclusion

The purpose of this scoping review was to map the available evidence on the individual and interpersonal determinants of food choice of athletes, examine the methods used for reporting determinants of food choice, report any relationship with demographic characteristics and diet quality or intake and report on the quality of studies. There were 15 studies that met the inclusion criteria, with an equal amount of qualitative and quantitative research design with variable quality of reporting. Methods employed were predominately semi-structured interviews and questionnaires for qualitative and quantitative studies, respectively. No longitudinal or intervention studies were found. The majority of studies have been published since 2018 and conducted across multiple countries with either mixed cohorts of athletes or focused on predominately endurance or team sports. Only one study focused specifically on female athletes. Most studies reported that performance and health were relevant to athlete food choice, with varying impact of competition season, the level of experience, sport culture, the cultural background or nationality, plus sex of the athlete, and the food environment. One study [29] reported on the relationship to diet quality and this was relevant to a single meal during competition.

The outcomes of this scoping review suggest that more research is needed on the multi-faceted determinants of food choice in athletes. Future research could explore the relationship between food choice, nutrition knowledge and diet quality or the change in food choice across the phase of the seasons and through injury and illness. Furthermore, qualitative methodology would be useful for better understanding of sex specific issues, in particular, those relevant to females. Use of validated measurement tools such as the AFCQ and robust reporting will enable critical interpretation of the study methods and outcomes for use in practice.

## Data Availability

Not applicable.

## Appendix

Search strategy.

SCOPUS (Elsevier B.V). Search conducted on 3rd August 2020.

**Table:**

Search	Query	Records retrieved
#1	*TITLE-ABS-KEY (*“food decision*" OR "food acceptance*" OR "food preference*" OR "food choice*" OR "food purchase" OR "food buy*" OR "determinants of eating" OR "determinants of nutrition")	30,900
#2	*TITLE-ABS-KEY ("athlet*" OR "sport*" OR "player*")*	4,57,363
#3	#1 AND #2	339
